# An Apolipoprotein A-I Mimetic Peptide Designed with a Reductionist Approach Stimulates Reverse Cholesterol Transport and Reduces Atherosclerosis in Mice

**DOI:** 10.1371/journal.pone.0068802

**Published:** 2013-07-09

**Authors:** Michael Ditiatkovski, Wilissa D’Souza, Rajitha Kesani, Jaye Chin-Dusting, Judy B. de Haan, Alan Remaley, Dmitri Sviridov

**Affiliations:** 1 Baker Heart and Diabetes Institute, Melbourne, Vic., Australia; 2 Lipoprotein Section, National Heart, Lung and Blood Institute, National Institutes of Health, Bethesda, Maryland, United States of America; The University of New South Wales, Australia

## Abstract

Apolipoprotein A-I (apoA-I) mimetic peptides are considered a promising novel therapeutic approach to prevent and/or treat atherosclerosis. An apoA-I mimetic peptide ELK-2A2K2E was designed with a reductionist approach and has shown exceptional activity in supporting cholesterol efflux but modest anti-inflammatory and anti-oxidant properties *in vitro*. In this study we compared these *in vitro* properties with the capacity of this peptide to modify rates of reverse cholesterol transport and development of atherosclerosis in mouse models. The peptide enhanced the rate of reverse cholesterol transport in C57BL/6 mice and reduced atherosclerosis in *Apoe^−/−^* mice receiving a high fat diet. The peptide modestly reduced the size of the plaques in aortic arch, but was highly active in reducing vascular inflammation and oxidation. Administration of the peptide to *Apoe^−/−^* mice on a high fat diet reduced the levels of total, high density lipoprotein and non-high density lipoprotein cholesterol and triglycerides. It increased the proportion of smaller HDL particles in plasma at the expense of larger HDL particles, and increased the capacity of the plasma to support cholesterol efflux. Thus, ELK-2A2K2E peptide reduced atherosclerosis in *Apoe^−/−^* mice, however, the functional activity profile after chronic *in vivo* administration was different from that found in acute *in vitro* studies.

## Introduction

Plasma levels of high density lipoprotein (HDL) inversely correlate with the incidence of cardiovascular disease and HDL is believed to protect against development of atherosclerosis. This well documented phenomenon prompted an advancement of various therapeutic approaches aimed at raising plasma levels of HDL as a treatment option and/or a preventative measure against the development of atherosclerosis [1]. Amongst those, the most direct approach is the intravenous infusion of HDL, usually in the form of reconstituted HDL (rHDL); a variation of this approach is the infusion of apolipoprotein A–I (apoA-I) mimetic peptides. In addition to the relatively low cost and simplicity of production, the advantages of apoA-I mimetic peptides over rHDL include ease of modification of their structure and function and availability of approaches allowing oral administration of the peptides (for review see [2]).

The original concept that led to the development of an apoA-I mimetic peptide was based on the assumption that the functional properties of apoA-I, the predominant apolipoprotein of HDL, are derived from its secondary structure, a series of 22-mer amphipathic α-helices linked by proline residues [3]. A number of apoA-I mimetic peptides were developed comprising of one or two helices mimicking the secondary structure of apoA-I, without sharing homology with the primary sequence of apoA-I. Tandem peptides (e.g. peptides containing two amphipathic α-helices coupled with a proline residue), although more costly, had superior apoA-I mimicking properties in relation to LCAT activation, cholesterol efflux and association with HDL [4]. Several peptides were tested in animal models of atherosclerosis and were found to be atheroprotective [5]–[9]. The mechanisms behind these atheroprotective effects however are not completely understood: in the majority of cases, infusion of the peptides minimally affected plasma levels of HDL cholesterol, yet improved various facets of HDL functionality. This makes it likely that after infusion the peptides may bind to plasma lipoproteins and act by affecting metabolism and functional properties of lipoproteins [10], [11]. These findings prompted the invention of more complex peptides (for review see [12]). For example, some peptides combined apoA-I-like sequences and those of the receptor-recognition domain of apolipoprotein E aiming to alter the metabolism of the lipoprotein particles that the peptide bound to [13]. Other modifications included the “stapling” of the peptides to stabilize their secondary structure [14].

In this study we explored an opposite approach, that of simplifying the peptide structure. A peptide design based on reductionist approach, an ELK peptide, was originally proposed by Fukushima et al [15]; it is comprised of three amino acids, glutamic acid, leucine and lysine, which were organized into a 22-mer with predicted secondary structure of a perfect type A1 amphipathic α-helix this structure is similar to the secondary structure of apoA-I helices. We have recently used this design approach to construct a series of unique peptides derived from the original ELK peptide and to investigate their structure-function relationships [16]. These peptides comprised of two 18-mer ELK peptides connected through a proline residue. By introducing modifications into the ELK peptide, we were able to create peptides displaying various anti-atherogenic activities *in vitro.* One of those peptides, ELK-2A2K2E, demonstrated an interesting combination of anti-atherogenic properties *in vitro*: exceptional activity and specificity in effecting cholesterol efflux from RAW 264.7 cells, as well as being active in reducing human monocyte activation [16]. At the same time, this peptide demonstrated a limited anti-oxidant capacity and limited capacity to inhibit VCAM-1 expression on endothelium. In this study, we used animal models to test the effect of a peptide with this unique combination of anti-atherogenic properties established *in vitro* on plasma lipoproteins, reverse cholesterol transport and several aspects of pathogenesis of atherosclerosis: accumulation of cholesterol, systemic and local inflammation and oxidation.

## Materials and Methods

### Ethics Statement

All experiments were approved by the Animal Ethics Committee of the Alfred Medical Research and Education Precinct (AMREP) and conformed to the Guide for the Care and Use of Laboratory Animals (NIH).

### Animal Model of Atherosclerosis

Male *Apoe^−/−^* (C57BL/6J-apoe^tm1Unc^) mice were obtained from the Alfred Medical Research and Education precinct (AMREP) Animal Services. At 10 weeks of age the animals were fed high fat diet (HFD) containing 21% fat and 0.15% cholesterol, available *ad libitum*. Synthesis and analysis of the ELK-2A2K2E peptide have been described previously [16]. ELK-2A2K2E was resuspended in PBS and administered to the animals intraperitoneally at a dose of 30 mg/kg three times a week for 4 or 16 weeks (n = 8 per time point); a control group (n = 8 per time point) received the same volume of PBS at the same frequency as the treatment group. The dose and frequency of injections were selected based on our previous study using apoA-I mimetic peptide 5A [7]. At the end of the treatment, mice were euthanized by CO_2_ inhalation and blood was collected by cardiac puncture. The aorta, up to the point of renal arteries, was removed for subsequent analysis. The heart was removed allowing for analysis of plaques within the aortic sinus region.

### Pharmacokinetics

The N terminus of the peptide was labelled with Alexa Flour 350 Succinimidyl Easter (Invitrogen) as described by Gaudriault and Vincent [17]. Pharmacokinetics of ELK-2A2K2E were examined in *Apoe^−/−^* mice following 2 weeks of HFD feeding to mimic the lipid profile of animals in the main study. Mice were injected with 1 mg of the labelled peptide intraperitonealy and approximately 40 µl of blood was collected from the tail vein of mice prior to the injection and after indicated periods of time post injection. Plasma peptide levels were determined for each time point on Victor plate reader (PerkinElmer) with 355 nm excitation and 450 nm emission wavelengths. The distribution of the peptide among lipoprotein fractions was assessed in 10 µl of plasma collected 2 h after peptide injection by FPLC using Superose 6 PC3.2/30 column (GE Healthcare). The distribution of cholesterol among lipoprotein fractions was assessed with Amplex Red Cholesterol Assay kit (Invitrogen).

### Histology

Aortae were perfused with PBS containing EDTA (2 mM) and excised from the animal. Aortae were cleaned of peri-aortic fat and stained for lipids with Sudan IV. Within each aorta, the stained area was quantified using Image J plus software.

Heart tissue containing the aortic sinus was embedded in optimal cutting temperature compound (OCT) (Tissue-Tek) and frozen for assessment of plaques within the aortic sinus region. Frozen tissue was cut on a Microme HM550 (Zeiss); 10 µm sections were collected when all three valves were apparent. Consecutive sections spanning 240 µm of the aortic sinus were cut and collected. Three sections per mouse, 80 µm apart, were stained with Oil Red O to determine lipid content within the lesion or Masson’s trichrome stain to detect collagen. Sections were also examined for macrophage content, as well as inflammatory and oxidation markers by immunohistochemistry. Briefly, following fixation in ice cold acetone, the sections were incubated with 3% H_2_O_2_, 10% serum and avidin/biotin blocking solution (Vector Laboratories). The sections were then incubated with a primary antibody (anti-CD68, AbD serotec; anti-VCAM-1, BD Pharmingen; Nitrotyrosine, Millipore) and a corresponding biotinylated secondary antibody. Staining was detected with Vectastain Elite ABC kit (Vector laboratories) and 3′3′-diaminobenzidine substrate (Sigma-Aldrich). Sections were counterstained with hematoxylin and Scotts water. Images were collected on Olympus FSX100 and quantified using ImagePro plus 6.0 software. Stained areas were expressed as a percentage of the total atheromatous plaque area.

### Analysis of Plasma Lipids and Lipoproteins

Blood collected from animals by submandibular bleeding at baseline and after 4 weeks of treatment, and by cardiac puncture after 16 weeks of treatment was centrifuged and plasma was collected. Plasma was subsequently analyzed for total cholesterol and triglyceride contents using colorimetric kits (Wako, Japan) according to the manufacturer’s instructions. To quantify HDL, ApoB containing particles were precipitated from the plasma by the dextran sulphate and magnesium chloride method [18] and the remaining solution analyzed for total cholesterol using a colorimetric kit. The size of the HDL particles was analyzed by non-denaturing gradient PAGE electrophoresis as described previously [19].

### Cholesterol Efflux Assay

Cholesterol efflux from RAW 264.7 cells was assessed as described previously [20]. Briefly, cellular cholesterol was labeled by incubation in serum-containing medium with [1α,2α (n)-^3^H]cholesterol (GE Health, final radioactivity 0.5 MBq/ml) for 48 h in a CO_2_ incubator. Cells were then washed and incubated for 18 h at 37°C in serum-free medium in the presence of the LXR agonist TO-901317 (4 µmol/L). Cells were washed and incubated for another 2 h at 37°C in serum-free medium containing 1% of whole mouse plasma and/or the peptide at a concentration of 10 µg/ml. Cholesterol efflux was expressed as the proportion of [^3^H]cholesterol transferred from cells to the medium. Non-specific efflux (i.e. the efflux in the absence of an acceptor) was subtracted.

### In Vivo Reverse Cholesterol Transport Assay

Reverse cholesterol transport *in vivo* was based on the assay developed by Zhang et al [21] with modifications as described previously [22]. Briefly, RAW 264.7 macrophage cells were simultaneously radiolabeled and loaded with cholesterol by incubation for 48 h with 29.7 mCi/ml [^3^H]cholesterol and acetylated LDL (50 µg/ml). Cells were washed, incubated for 24 h in serum-free medium, and resuspended in 0.15 M sterile saline at a concentration of 10^7^ cells/ml. Cells were injected intraperitoneally into male C57BL/6 mice (2×10^6^ cells containing 5×10^6^ dpm per mouse) (*n* = 7). Mice were injected intraperitoneally with vehicle, 5A or ELK-2A2K2E (30 mg/kg) twice: 8 h prior and immediately before cell injection. After 24 h, mice were euthanized, and blood, liver and feces were collected. Aliquots of plasma were counted, cholesterol from liver and feces was extracted, and cholesterol was separated by thin-layer chromatography and counted.

### Statistics

Data is shown as means ±SEM. Statistical significance of the differences was assessed by *t*-test or Mann-Whitney U test on ranks when data did not follow a normal distribution. *Ex vivo* assays were done in quadruplicate.

## Results

### The Peptide

The sequence of the ELK-2A2K2E peptide is: EKLKAKLEELKAKLEELL-P-EKLKAKLEELKAKLEELL. This peptide is comprised of two type A1 α-helices connected via proline. It has an overall neutral charge and mean hydrophobicity of −0.47 (Kyte & Doolittle) and 180^o^ hydrophobic face. It has been derived from the original ELK peptide by introducing the following substitutions: A for E in positions 5 and 12, K for L in positions 6 and 11 and E for K in positions 9 and 15. The functional properties of this peptide have been described previously and are characterized by an exceptional capacity to support ABCA1-specific cholesterol efflux and modest capacity to inhibit inflammation and oxidation [16].

### Pharmacokinetics

**Figure 1:**
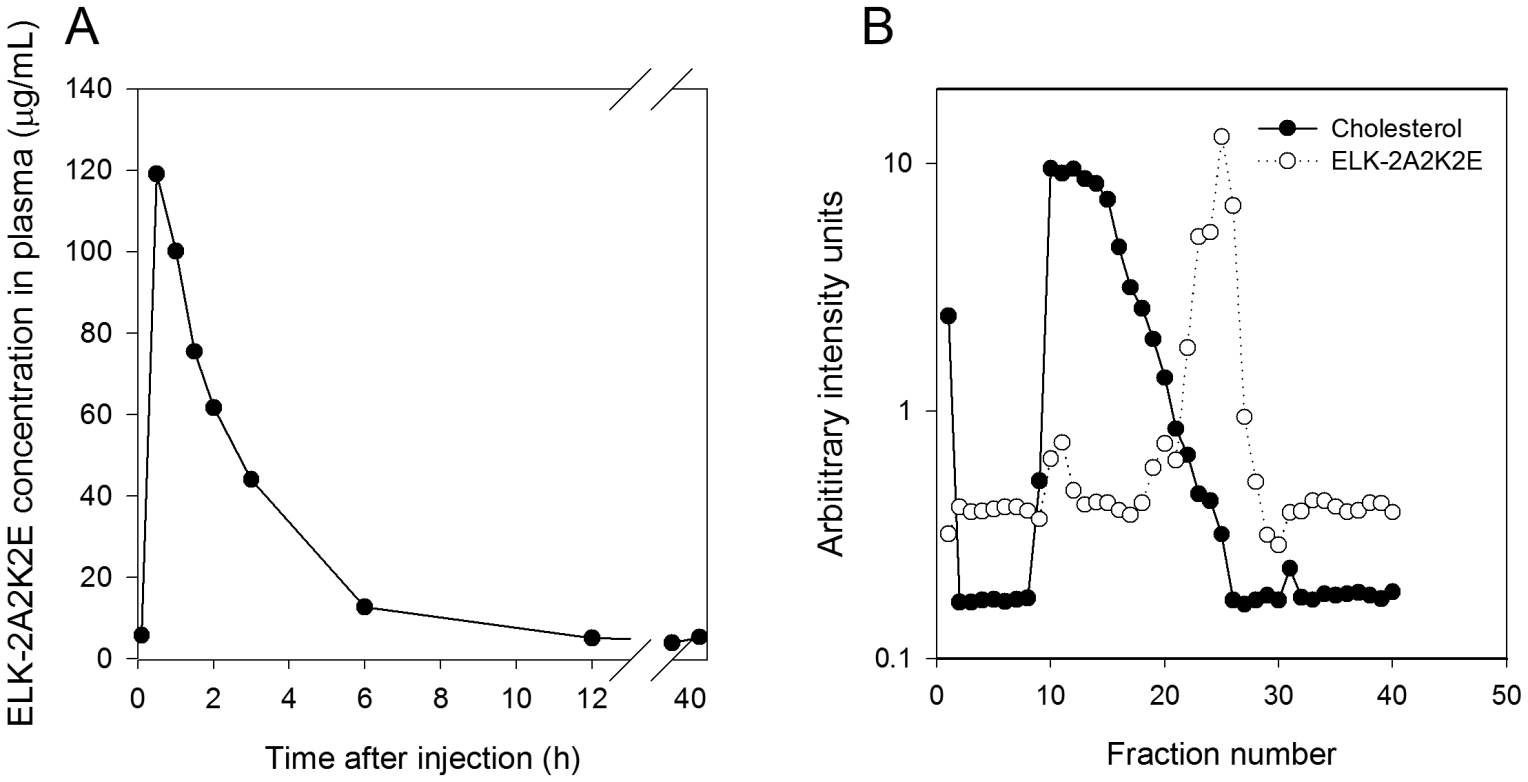
Pharmacokinetics and distribution of ELK-2A2K2E in mouse plasma. A – Pharmacokinetics of ELK-2A2K2E in *Apoe^−/−^* mice fed high- fat diet (representative curve from one animal). B – FPLC analysis of distribution of ELK-2A2K2E and cholesterol in plasma collected 2 h after peptide injection.

To investigate pharmacokinetics of ELK-2A2K2E, 1 mg of the Alexa 350-labelled peptide was injected intraperitoneally into *Apoe^−/−^* mice kept on high fat diet for 14 days; the time-course of the peptide concentration in plasma is shown in Fig. 1A. It was established in a preliminary experiment using mass spectrometry that the label remained attached to the peptide after exposure to plasma *in vitro* and *in vivo* (not shown). The plasma concentration of the peptide peaked at approximately 120 µg/ml 30 min after injection; half-life of the peptide was estimated at 2.25 h. However, a proportion of the peptide (approximately 4% of maximal concentration or 5 µg/ml) remained in the plasma for at least 48 h (Fig. 1A). The distribution of the labelled peptide in mouse plasma was analysed by FPLC in plasma samples taken 2 h after injection of the peptide (Fig. 1B). At this time point, a majority of the peptide remained free with a proportion of the peptide bound to both HDL and LDL fractions. It must be recognized however that these experiments utilized a derivatized peptide; attaching label to the peptide may have affected its properties and this needs to be taken into account when interpreting the data.

### Development of Atherosclerosis

**Figure 2:**
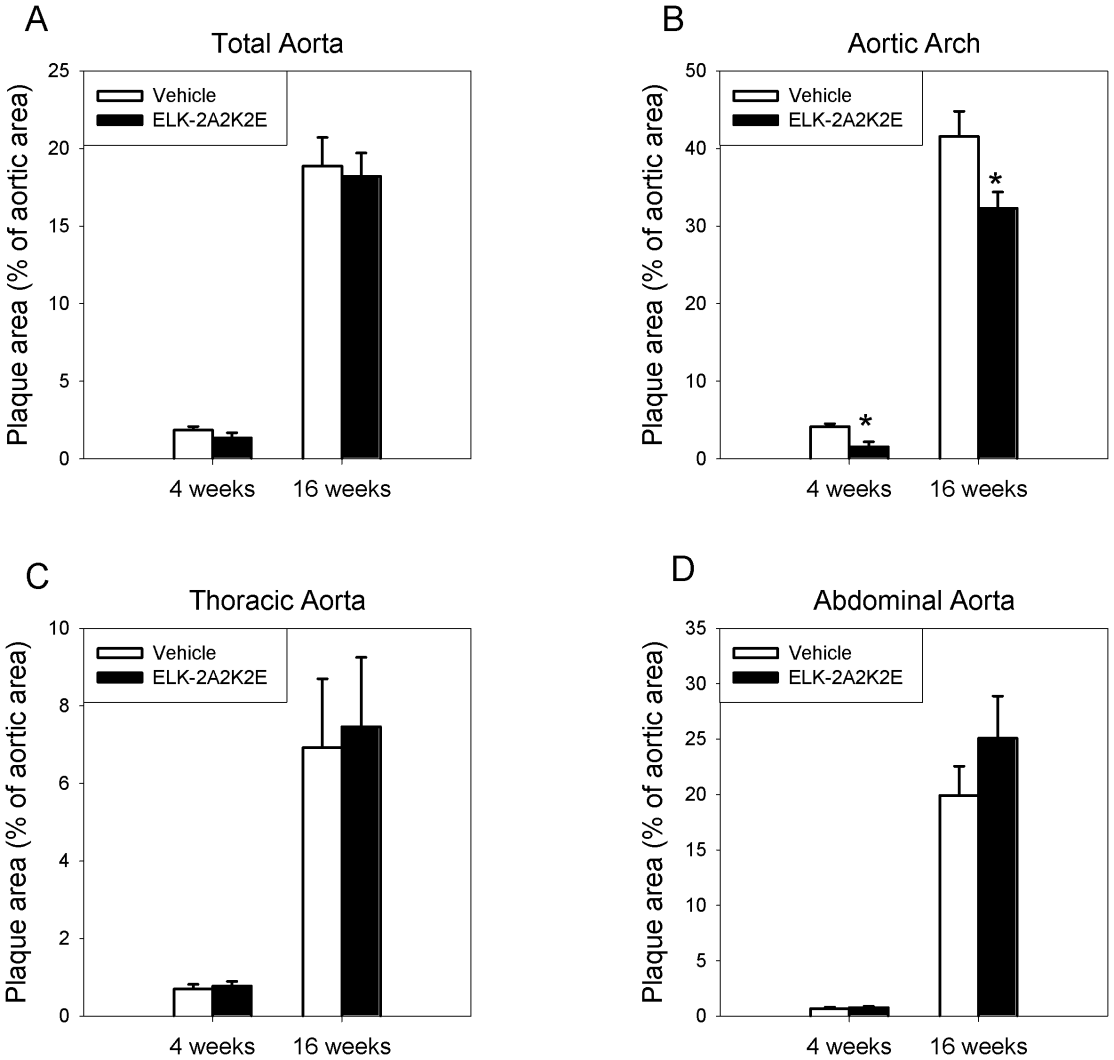
Effect of ELK-2A2K2E treatment on development of atherosclerosis: *en face* analysis after staining with Sudan IV. **A -** Percentage of atherosclerotic lesions in the total aorta; n = 8 for each bar. **B -** Percentage of atherosclerotic lesions in the aortic arch; *p<0.03; **p<0.05 *versus* vehicle; n = 8 for each bar. **C-** Percentage of atherosclerotic lesions in the thoracic aorta; n = 8 for each bar. **D -** Percentage of atherosclerotic lesions in the abdominal aorta; n = 8 for each bar. Percentages were calculated as an area stained with Sudan IV divided by total area.

Male *Apoe^−/−^* mice were kept on a high fat diet for 4 or 16 weeks with thrice weekly intraperitoneal injections of either peptide ELK-2A2K2E (30 mg/kg) or vehicle (PBS). There was a slight reduction in percent of area occupied by plaques in the whole aorta in the ELK-2A2K2E group compared to the vehicle group at both time points after *en face* analysis, however the difference did not reach statistical significance (Fig. 2A). When lesions in the aortic arch, the area most susceptible for the development of atherosclerosis in this model, were analysed, animals treated with ELK-2A2K2E showed a reduction of 63% (p<0.005) after 4 weeks on HFD and of 31% (p<0.03) after 16 weeks on HFD compared to the corresponding vehicle group (Fig. 2B). There was no statistically significant difference in the area occupied by atherosclerotic lesions in the thoracic (Fig. 2C) and abdominal (Fig. 2D) regions of aorta between treatment and vehicle groups at both time points. Despite regional differences we do not believe that treatment had a site-specific effect; rather, the effect of treatment was only detectable in the region most susceptible for atherosclerosis.

**Figure 3:**
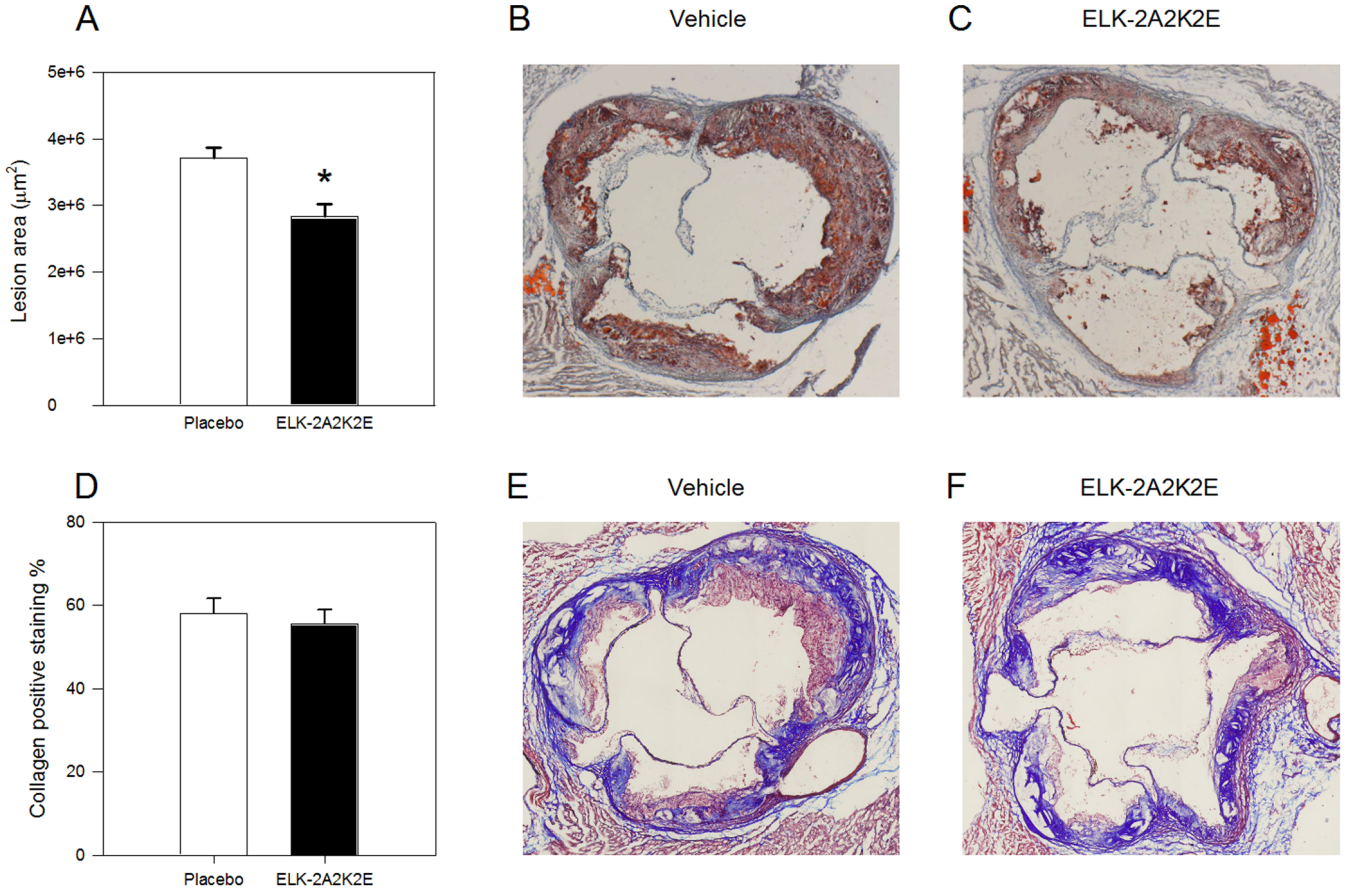
Effect of ELK-2A2K2E treatment on development of atherosclerosis: histological analysis. **A-** Quantitation of the area occupied by lesions within the aortic sinus region after staining with Oil Red O; *p<0.02 *versus* vehicle; n = 8 for each bar. **B -** Representative section of an aortic sinus from a control mouse stained with Oil Red O. **C -** Representative section of an aortic sinus from a mouse treated with ELK-2A2K2E stained with Oil Red O. **D -** Analysis of the abundance of collagen within the aortic sinus region after staining with Masson’s trichrome stain; n = 8 for each bar. Values represent collagen positive staining as a percentage of lesion area. **E -** Representative section of an aortic sinus from a control mouse stained with Masson’s trichrome stain. **F -** Representative section of an aortic sinus from a mouse treated with ELK-2A2K2E stained with Masson’s trichrome stain.

The detailed analysis of atherosclerotic plaques was undertaken on cryosections of the aortic sinus region after 16 weeks on HFD. We analysed the region with most pronounced atherosclerosis because this provided a better opportunity to discover differences in the morphology of the plaque. Also, this region can be precisely mapped ensuring that exactly the same parts of the vessel were compared, thus greatly reducing variability. The severity of atherosclerosis was assessed by staining sections with Oil Red O. In mice treated with ELK-2A2K2E the average lesion area was reduced by 38% (p<0.02) compared to the vehicle group (Fig. 3A). Figs. 3B, C show representative sections of Oil Red O staining of the aortic sinus region of mice treated with vehicle (Fig. 3B) and ELK-2A2K2E (Fig. 3C); larger plaques were found in the vehicle group.

Abundance of collagen in atherosclerotic plaque is an important measure of plaque stability; HDL was shown to increase abundance of collagen in mouse model of atherosclerosis [23]. However we found no statistically significant difference in the abundance of collagen between mice treated with ELK-2A2K2E and vehicle (Fig. 3 D–F).

**Figure 4:**
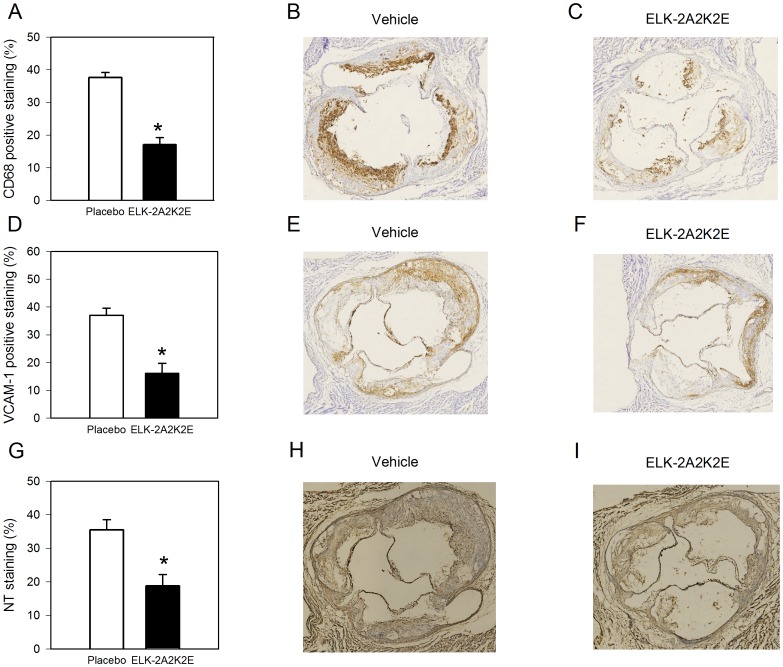
Effect of ELK-2A2K2E treatment on inflammation and oxidation in aorta. **A -** Analysis of macrophage infiltration within the aortic sinus region after staining with anti CD68; *p<0.001 *versus* vehicle; n = 8 for each bar. **B -** Representative section of an aortic sinus from a control mouse stained with anti CD68. **C -** Representative section of an aortic sinus from a mouse treated with ELK-2A2K2E stained with anti CD68. **D** **-** Analysis of the abundance of VCAM-1 in sections from the aortic sinus region; *p<0.001 *versus* vehicle; n = 8 for each bar. **E -** Representative section of an aortic sinus from a control mouse stained with anti VCAM-1. **F -** Representative section of an aortic sinus from a mouse treated with ELK-2A2K2E stained with anti VCAM-1. **G -** Analysis of the abundance of nitrotyrosine (NT) in sections from the aortic sinus region; *p<0.001 *versus* vehicle; n = 8 for each bar. **H -** Representative section of an aortic sinus from a control mouse stained with anti- nitrotyrosine. **I -** Representative section of an aortic sinus from a mouse treated with ELK-2A2K2E stained with anti-nitrotyrosine. Values represent positive staining with the corresponding antibody as a percentage of lesion area.

The level of inflammation within the plaques was assessed by evaluating the area of the lesion occupied by macrophages (defined as CD68-positive cells). The abundance of macrophages in the lesions of mice treated with ELK-2A2K2E was 2.4-fold lower (p<0.001) compared to the vehicle group (Fig. 4A). Figs. 4B, C show representative immunohistochemical staining with anti-CD68 of sections from the aortic sinus region of mice treated with vehicle (Fig. 4B) and ELK-2A2K2E (Fig. 4C); significantly greater infiltration of macrophages was found in the vehicle group.

Another element of inflammation relevant to the pathogenesis of atherosclerosis is the abundance of adhesion molecules on the surface of vascular cells. The abundance of VCAM-1 (percentage of area stained with anti-VCAM-1 antibody) was 2.3-fold lower in the lesions of mice treated with ELK-2A2K2E (p<0.001) compared to the vehicle group (Fig. 4D). Figs. 4E, F show representative immunohistochemical staining with anti-VCAM-1 antibody of sections from aortic sinus region of mice treated with vehicle (Fig. 4E) and ELK-2A2K2E (Fig. 4F); significantly greater abundance of VCAM-1 staining was observed in the vehicle group.

An important element of pathogenesis of atherosclerosis is the enhanced abundance of oxidation products in the plaque. We used nitrotyrosine as a marker of protein oxidation; the amount of nitrotyrosine (percentage of area stained with anti-nitrotyrosine antibody) in the lesions of mice treated with ELK-2A2K2E was 1.8-fold lower (p<0.001) compared to the vehicle group (Fig. 4G). Figs. 4H, I show representative immunohistochemical staining with anti-nitrotyrosine antibody of sections from the aortic sinus region of mice treated with vehicle (Fig. 4H) and ELK-2A2K2E (Fig. 4I); significantly greater abundance of nitrotyrosine staining was found in the vehicle group.

### Plasma Lipoproteins

**Figure 5:**
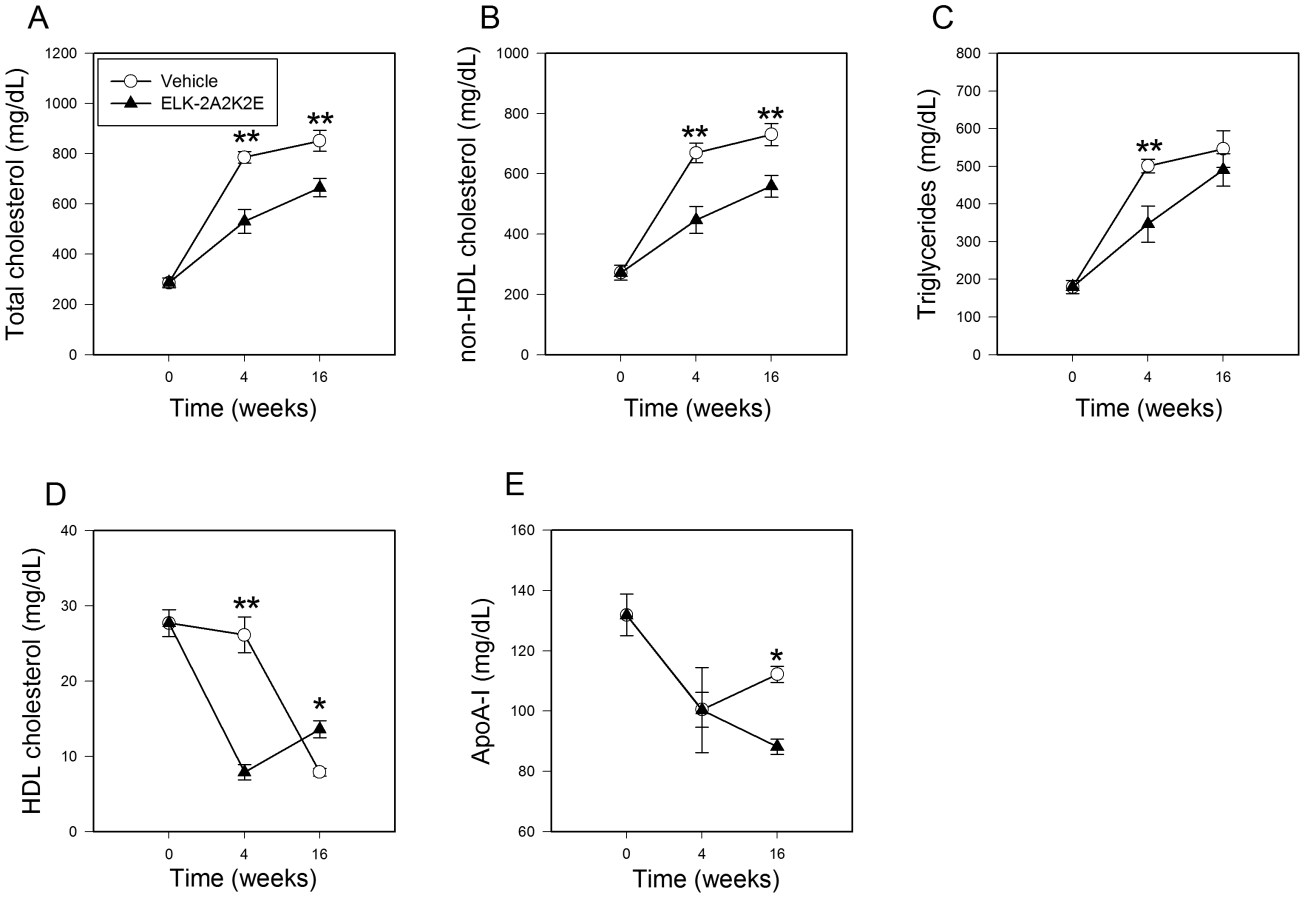
Effect of ELK-2A2K2E on plasma lipids and lipoproteins. **A -** Total cholesterol; **B -** non-HDL cholesterol; **C** - Triglycerides; **D -** HDL cholesterol; **E** **-** apoA-I. *p<0.05, **p<0.01 *versus* vehicle; n = 8 for each point.

Dynamic changes in plasma lipids after initiation of high-fat diet are shown in Fig. 5. Total cholesterol (TC) content rose in both groups after initiation of high fat diet, but while in the vehicle group the TC content nearly tripled, in mice treated with ELK-2A2K2E it doubled, resulting in TC content in treated mice being 1.8-fold lower compared to the vehicle group (Fig. 5A). Changes in TC were closely followed by changes in cholesterol in non-HDL cholesterol (non-HDL-C) (Fig. 5B) and triglycerides (TG) (Fig. 5C); both non-HDL-C and TG rose with their concentration being approximately 1.5-fold higher in the vehicle group compared to the ELK-2A2K2E group after 4 weeks of treatment. In contrast, the plasma levels of both HDL cholesterol (HDL-C) and apoA-I fell after initiation of high fat diet (Fig. 5D, E). The fall was greater in the ELK-2A2K2E-treated group and as a result, the plasma levels of HDL-C and apoA-I were lower in the ELK-2A2K2E-treated group.

### HDL Size and Functionality

**Figure 6:**
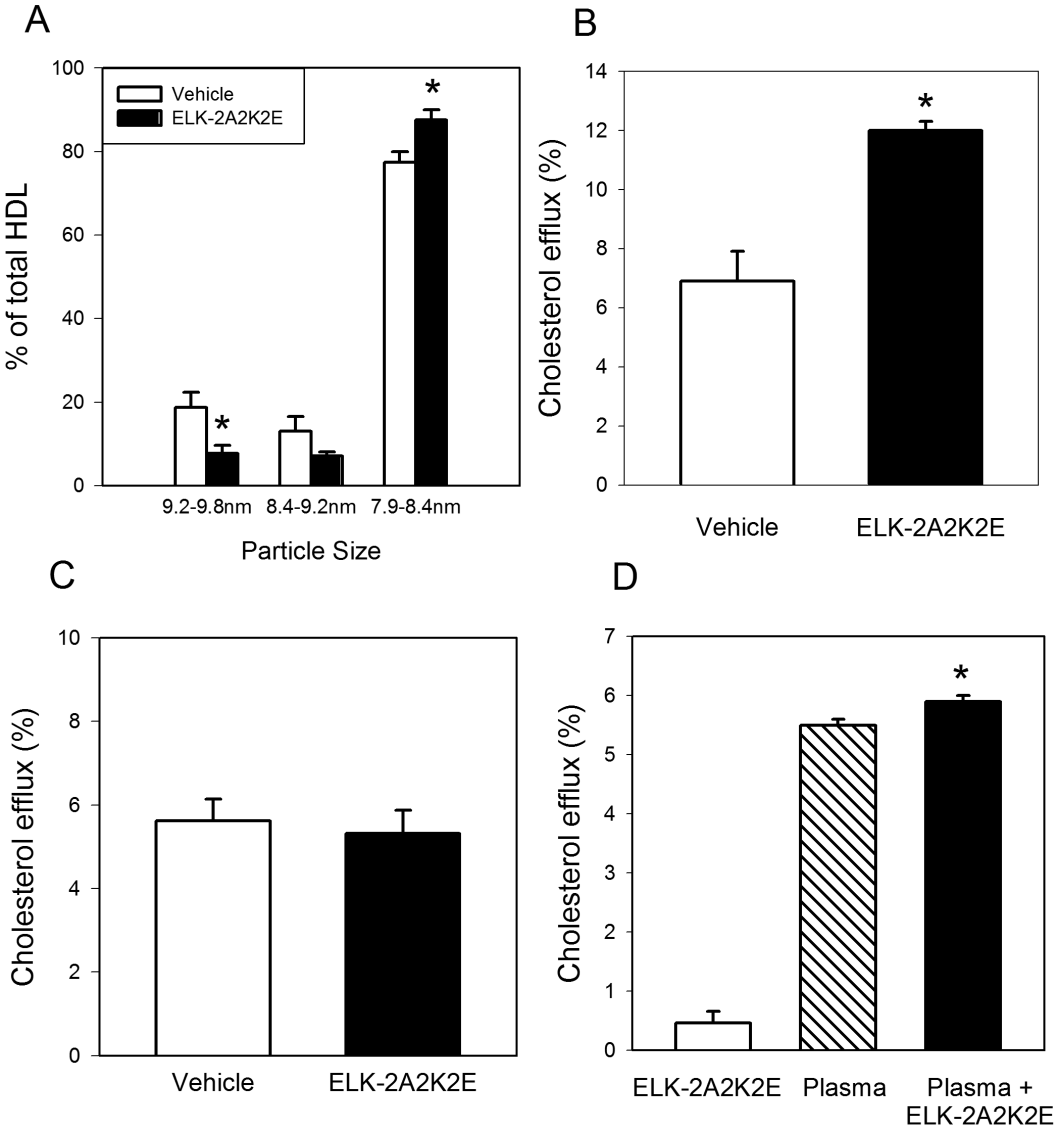
Effect of ELK-2A2K2E on HDL size distribution and HDL functionality. **A -** Analysis of size distribution of HDL particles in two groups of mice assessed by non-denaturing electrophoresis; *p<0.02 *versus* vehicle. **B -** Cholesterol efflux to plasma (final concentration 1%) from two groups of mice; plasma collected 2 h after peptide injection. **C -** Cholesterol efflux to plasma (final concentration 1%) from two groups of mice; plasma collected 72 h after peptide injection. **D** **-** Efflux of cholesterol to plasma (final concentration 1%), ELK-2A2K2E (final concentration 10 µg/ml) or a combination of the two. *p<0.05 *versus* plasma.

Next we tested the size and functionality of HDL in the plasma from the two groups of animals. The size of HDL was assessed in plasma samples collected three days after the last injection of the peptide using non-denaturing electrophoresis followed by Western blotting with anti-apoA-I antibodies. Most of the HDL particles in mouse plasma were 8.2 nm in diameter with two minor fractions with average diameter of 8.8 nm and 9.5 nm. The proportion of smaller particles was higher while the proportion of larger particles was lower in plasma of ELK-2A2K2E-treated mice compared to the vehicle group; there was no statistically significant change in the proportion of medium-sized particles (Fig. 6A).

We then tested plasma samples in an *ex vivo* cholesterol efflux assay at a concentration of 1%. When plasma samples collected 2 h after injection of the peptide or vehicle were tested, the efflux to plasma of treated animals was almost double the efflux to plasma of untreated animals (Fig. 6B). However no statistically significant difference was found between the treatment and vehicle groups when plasma collected 72 h after injection was tested (Fig. 6C). When we added ELK-2A2K2E to mouse plasma *in vitro* to the final concentration of 10 µg/ml in 1% plasma, there was a modest but statistically significant increase in the capacity of plasma to effect cholesterol efflux compared to plasma alone (Fig. 6D). The increase was similar to combination of the efflux to both plasma and peptide added separately and much smaller that the increase found in vivo. These findings indicate that cholesterol efflux properties of the peptide are intact and add to the cholesterol efflux capacity of plasma either by binding to lipoproteins or on its own.

### Reverse Cholesterol Transport

**Figure 7:**
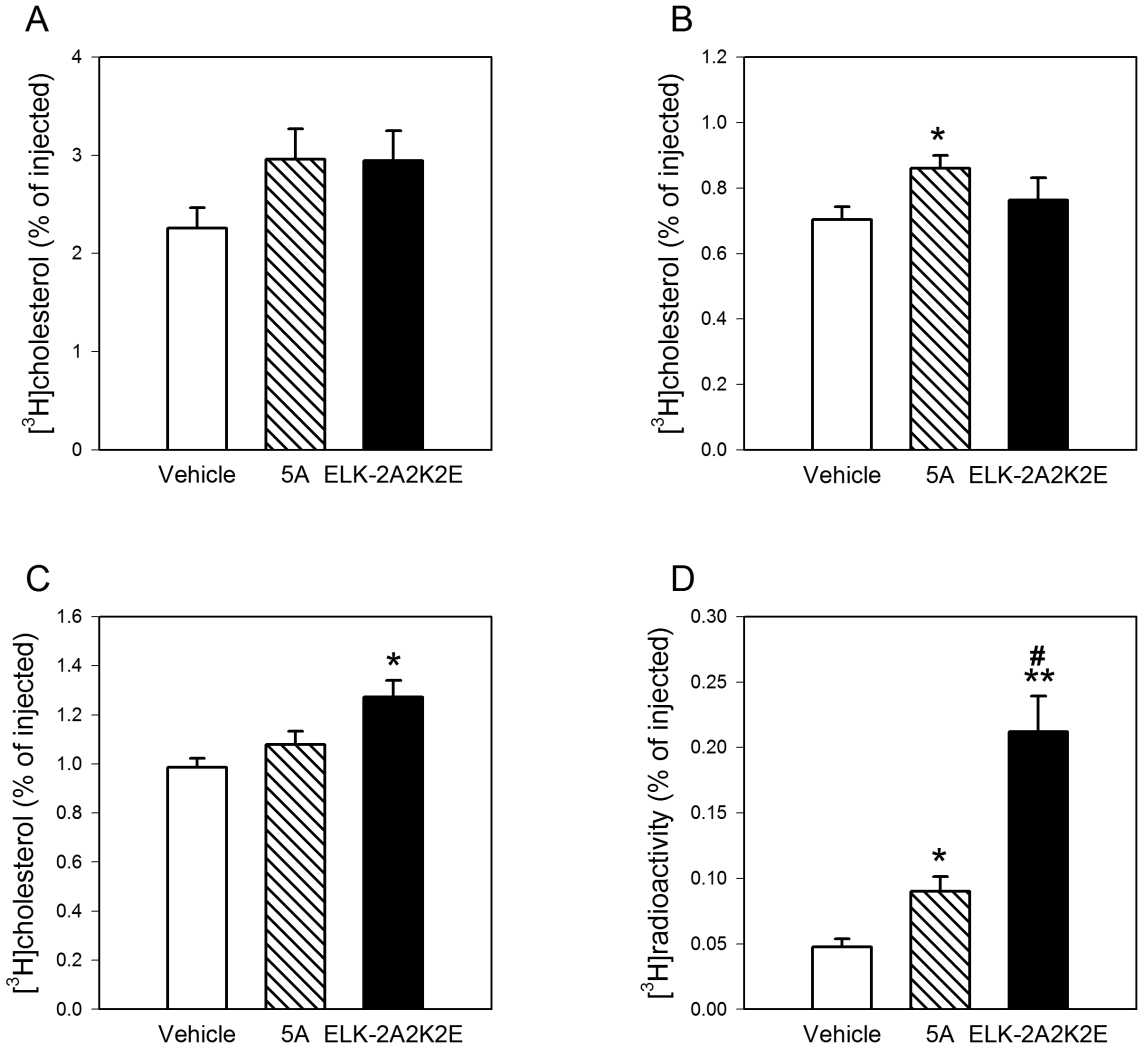
Effect of ELK-2A2K2E on the rate of reverse cholesterol transport *in vivo.* RAW 264.7 macrophages were labeled with [^3^H]cholesterol, loaded with AcLDL and transplanted into the intra-peritoneal cavity of C57Bl/6 mice. Proportion of [^3^H]cholesterol out of total injected in macrophages recovered after 24 h in plasma (**A**), liver (**B**), fecal cholesterol (**C**) and fecal bile acids (**D**) are shown; *p<0.05; **p<0.001 *versus* vehicle; ^#^p<0.01 *versus* 5A; n = 7 for each bar.

We also tested the effectiveness of the ELK-2A2K2E peptide in a well established animal model of reverse cholesterol transport [22]. We have chosen C57BL/6 mice for these experiments to avoid confounding effects of dyslipidemia in *Apoe^−/−^* mice. In these experiments we compared ELK-2A2K2E with vehicle and another very effective apoA-I mimetic peptide, 5A [7], [16], [24]. 5A was previously shown to be effective in supporting RCT in vivo [7], however, unlike testing the effects on the development of atherosclerosis, the methodology only allows for direct comparison when compounds are tested side-by-side. Peptides were injected into mice intraperitoneally in lipid-free form followed by injection of [^3^H]cholesterol-labeled, cholesterol-loaded macrophages, and egress of labeled cholesterol to plasma, liver and feces was monitored after 24 h. Both 5A and ELK-2A2K2E caused a tendency for an elevation in the amount of labeled cholesterol in plasma, but the difference with vehicle was not statistically significant (Fig. 7A). Peptide 5A caused statistically significant elevation of the amount of labeled cholesterol in the liver (p = 0.02); however there was no difference between the vehicle group and the group treated with ELK-2A2K2E (Fig. 7B). In contrast, when fecal cholesterol was analyzed, there was a statistically significant difference between the group treated with ELK-2A2K2E and the vehicle (p<0.005) and 5A group (p<0.05) groups, but there was no statistically significant difference between the vehicle group and the 5A group (Fig. 7C). Both peptides caused a significant elevation in the amount of labeled cholesterol excreted as fecal bile acids: compared to the vehicle group, 2- and 4- fold more labeled cholesterol was found in the fecal bile acids in the groups treated with 5A and ELK-2A2K2E respectively (p<0.01); ELK-2A2K2E was more effective compared to 5A (p<0.01) (Fig. 7D). The differences between ELK-2A2K2E and 5A indicate that they may not differ in cholesterol efflux properties, but ELK-2A2K2E may be more effective in delivering cholesterol to the liver pool destined for excretion and conversion to bile acids. Thus, both peptides caused only a small elevation in the amount of labeled cholesterol in transitory RCT cholesterol pools, plasma and liver, with greater effects on accumulation of labeled cholesterol in the cumulative end pool, feces.

## Discussion

This study investigated the effects of a novel apoA-I mimetic peptide, ELK-2A2K2E, on development of atherosclerosis, lipoprotein metabolism and efficiency of reverse cholesterol transport in animal models. As expected, injection of the peptide inhibited the development of atherosclerosis in *Apoe^−/−^* mice and enhanced reverse cholesterol transport in C57BL/6 mice. However, investigation of the mechanisms responsible for these effects led to unexpected outcomes. Given that ELK-2A2K2E was exceptionally effective in supporting cholesterol efflux *in vitro* [16], we expected that ELK-2A2K2E would also be very effective in reducing plaque lipid content, but it appears, that this *in vitro* property of the peptide has not translated into *in vivo* efficiency. The observed effects on lipid accumulation in aortic sinus (38% reduction) were similar to those observed for similar doses of several other apoA-I mimetic peptides (30–45% reduction of lipid accumulation) [6]–[8], [25]–[30], although this was less than the effect shown for orally introduced high dose of peptide D4F (70% inhibition) [5]. It must be noted that a majority of these studies used female mice, while male mice were used in this study and that the group sizes were bigger in most other studies; both factors may affect the comparison. Conversely, the peptide was only modestly active in reducing VCAM-1 expression on endothelium and in preventing LDL oxidation *in vitro* [16], and yet it was remarkably effective in reducing VCAM-1 abundance and accumulation of oxidation products *in vivo*. In our study the peptide induced long-term reductions in plasma levels of all lipoproteins and changes in the size of HDL. It appears that chronic effects of peptide injection are not a simple reflection of its presence in lipoprotein particles, but are a consequence of a delayed and more complex metabolic effect. The capacity of mouse plasma to effect cholesterol efflux was increased by the peptide. In the *in vivo* model of reverse cholesterol transport, both ELK-2A2K2E and another peptide, 5A, mainly affected accumulation of cholesterol in feces, but not in plasma. This suggests that properties and metabolism of lipoproteins, not just cholesterol efflux, are affected by the peptides. Collectively, these findings support the hypothesis that the mechanism of action of ELK-2A2K2E is not a simple supplementation of the anti-atherogenic activities of HDL with additional activity of its own, but instead changes in properties of the lipoprotein particles, a hypothesis also supported by the observed changes in the size of HDL particles. It must be recognized however that HDL represents a minor lipoprotein fraction in apoE^−/−^ mice fed with HFD. Our findings are consistent with several observations that mimetic peptides have a major effect on the functionality of the lipoproteins they bind to [10], [11], [13] or even on exchange of constituencies between LDL and HDL [31]. An alternative explanation is that peptides reduce local production of metabolites of arachidonic and linoleic acids in the intestine as was suggested by Navab et al [32], [33], a possibility especially relevant for the experiments with intraperitoneal route of administration. A limitation of this study is that we did not evaluate the effect of the peptide on the number of monocytes in the plasma that could be influenced by the peptide [34] and to contribute to the observed effects. Also, the effects were only tested in one animal model; the responses may be different in e.g. LDLR^−/−^ mice. Our findings underline the complexity of translating *in vitro* properties of the peptides into *in vivo* outcomes.

An intriguing finding is that the substantial reduction in the number of macrophages and oxidation products caused by the peptide was not translated into a proportional reduction in the size of atherosclerotic plaques. This finding raises the question of why atherosclerosis progresses against a background of markedly reduced inflammation and oxidation, and more generally, what elements of atherosclerosis pathogenesis are essential for its progression? Further studies with peptides that specifically affect individual aspects of pathogenesis of atherosclerosis are required to clarify these issues.

Injection of the peptide caused significant reductions of cholesterol in all lipoprotein fractions. A most likely explanation for this finding is that lipoprotein particles carrying the peptide have a faster catabolic rate; however this was not examined directly. Because HDL is only a minor lipoprotein fraction in *Apoe^−/−^* mice maintained on a high fat diet, the implication of this finding is that the peptide binds to all lipoprotein fractions, not just HDL. This is consistent with *ex vivo* experiments with peptide 5A [7] and other apoA-I mimetic peptides [35] and was demonstrated directly in this study when distribution of the peptide among plasma constituencies was assessed. Reduction of plasma cholesterol content would have a major contribution to the anti-atherogenic effect of the peptide, especially if it results from faster clearance of lipoproteins. Unfortunately hyperlipidemic animal models of atherosclerosis do not provide an opportunity to evaluate relative contribution of reducing plasma cholesterol and of improving various anti-atherogenic facets of HDL when the two coincide.

A number of apoA-I mimetic peptides have been developed over the years and several of them, variants of 4F [5], [6], [25], [26], [28], [29], 3F [27], 6F [30], ATI-5261 [8], mR18L [36] and 5A [7], have been tested in animal models of atherosclerosis. Only lipid accumulation has been evaluated in all, but one [25] of these studies thus making it difficult to assess the effect of the peptides on individual facets of atherosclerosis, and consequently to fully understand the mechanisms of their action. In our study, we evaluated the effect of an ELK peptide on several facets of atherosclerosis; this approach not only provides mechanistic insight, but also addresses the issue of relationship between properties of peptides *in vitro* and *in vivo*. Further, ELK peptides have a number of advantages over existing peptides. The very simple structure makes them more likely to have a unique mechanism and a favourable profile of side effects. The simplicity of the structure of these peptides is an advantage in making predictions on the effects of further structural modification on their properties *in vitr*o and *in viv*o. They may represent a simple platform to build peptides with various functional properties targeting specific facets of atherosclerosis and adjusting their pharmacological properties (e.g. adjusting life-time or and making them suitable for oral administration). This makes derivatives of the ELK peptides attractive for further development of the peptides both as a research tool and potentially as an anti-atherosclerosis therapy.
